# New Insights Into the Role of Inflammation in the Brain in Heart Failure

**DOI:** 10.3389/fphys.2022.837723

**Published:** 2022-03-03

**Authors:** Emilio Badoer

**Affiliations:** School of Health and Biomedical Sciences, RMIT University, Melbourne, VIC, Australia

**Keywords:** inflammation, central nervous system, heart failure, nanoparticles, TNF, interleukins

## Abstract

Heart failure is a growing medical problem. Although the underlying aetiology of heart failure differs according to the phenotype, there are some common characteristics observed in patients with heart failure. These include an increased sympathetic nerve activity, an activated renin–angiotensin system, and inflammation. The mechanisms mediating the increased sympathetic activity are not completely understood but the central nervous system plays a major role. Activation of the renin–angiotensin system plays an active role in the remodelling of the heart and in fluid and electrolyte imbalance. The presence of a central renin–angiotensin system means that locally produced angiotensin in the brain may also play a key role in autonomic dysfunction seen in heart failure. Markers of inflammation in the heart and in the circulation are observed in patients diagnosed with heart failure. Circulating pro-inflammatory cytokines can also influence cardiac function further afield than just locally in the heart including actions within the brain to activate the sympathetic nervous system. Preclinical evidence suggests that targeting the pro-inflammatory cytokines would be a useful therapy to treat heart failure. Most clinical studies have been disappointing. This mini-review suggests that pro-inflammatory cytokines in the brain play a key role and there is a problem associated with access of effective doses of the drugs to the site of action in the brain. The recent advances in nanotechnology delivery techniques may provide exciting future technology to investigate the role of specific pro-inflammatory mediators as novel targets within the brain in the treatment of heart failure.

## Introduction

### Heart Failure Is a Growing Problem

Heart failure is a growing medical problem. Although some studies had suggested that the growth in heart failure cases had plateaued, more recent evidence shows that the worldwide incidence of heart failure continues to grow, particularly in the elderly citizens of our community (Roth et al., 2015; Conrad et al., 2018; Cleland et al., 2019). The cost to the health system of caring for patients with heart failure is huge and in the United States alone, the total cost has been estimated to be over $30 billion annually, an extraordinary amount (Bozkurt et al., 2021b). By world standards, the fastest rate of rise in the prevalence of heart failure is occurring in China and India (Bragazzi et al., 2021). Given the population of these two countries, the future global health burden of heart failure is starkly clear.

### Phenotype of Heart Failure

Heart failure, as the name suggests, is a functionally abnormal heart that results from either structural and/or functional deficits. These induce an elevation in intracardiac pressures and/or reduced cardiac output. The level of ejection fraction has normally determined the phenotype of heart failure and the latest terminology that has been suggested defines three over-arching phenotypes; (i) heart failure with reduced ejection fraction (HFrEF) where ejection fraction is less than 40%, (ii) heart failure with mildly reduced ejection fraction (HRmEF) where ejection fraction is between 41 to 49%, (iii) heart failure with preserved ejection fraction (HFpEF) where ejection fraction is greater than 50% (Bozkurt et al., 2021a). The majority of patients diagnosed with heart failure have ejection fractions less than 50% (i.e., HFrEF or HFmEF). Generally, patients with HFrEF are believed to have worse prognosis but not all studies provide evidence for this view (Pocock et al., 2013). Accurate diagnosis of the phenotype of heart failure is important in guiding therapy. Pharmacological treatment of patients with HFrEF is more successful than in patients with HFpEF where convincing evidence of reduced mortality and morbidity is somewhat scarce. Some recent trials suggest there may be a benefit (Solomon et al., 2016, 2020).

### Characteristics of Heart Failure: Increased Sympathetic Nerve Activity

Although the underlying aetiology of heart failure differs according to the phenotype, as suggested by the different responses to pharmacological treatment, there are some common characteristics observed in patients with heart failure. These include an increased sympathetic nerve activity, an activated renin–angiotensin system, and inflammation. Why, then, does pharmacological therapy improve HFrEF with relatively little impact on HFpEF?

**Figure 1:**
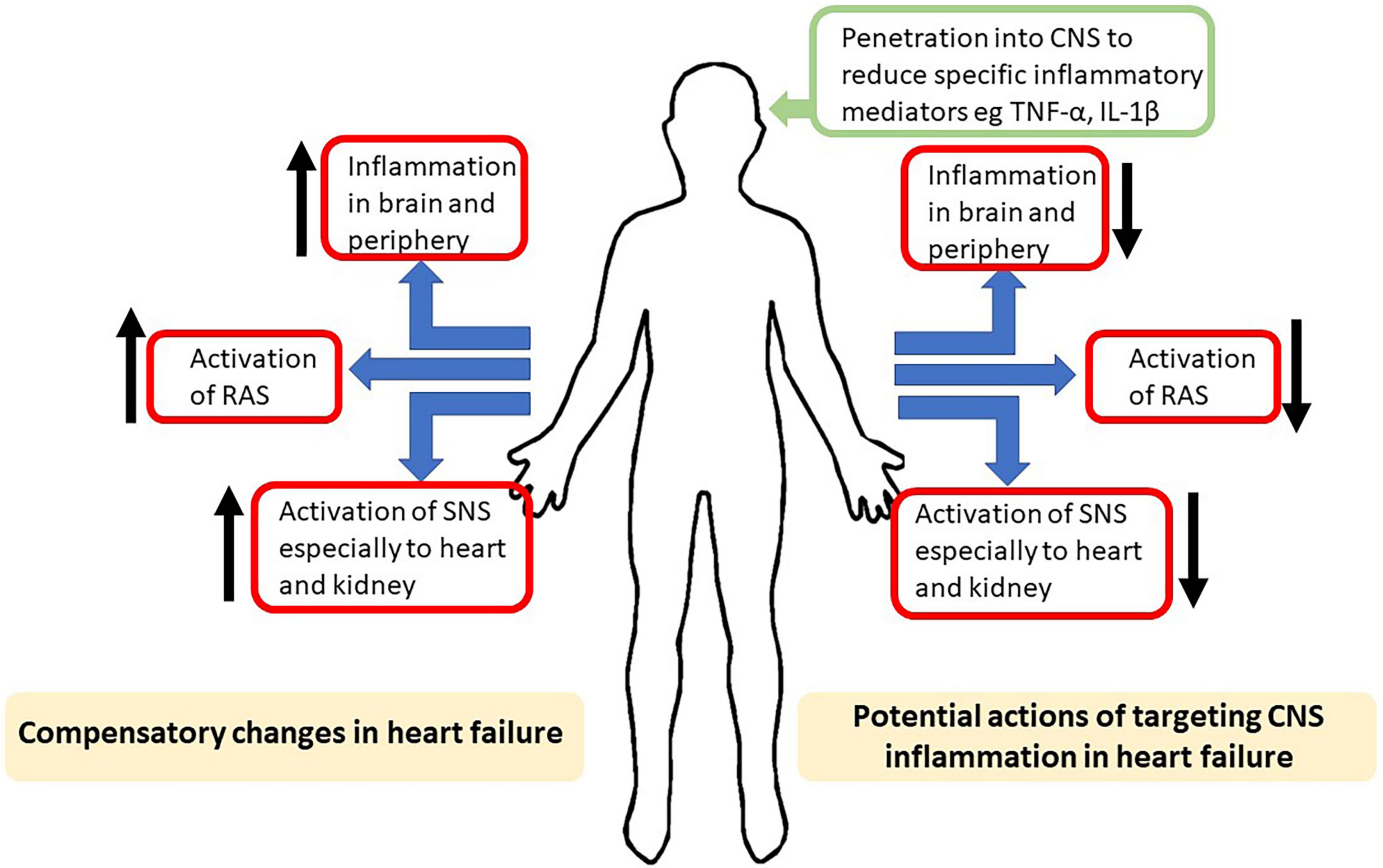
Schematic highlighting, on the left side, the compensatory increases in inflammation, activation of the renin–angiotensin system (RAS) and activation of sympathetic nerve system (SNS) induced by heart failure. On the right is shown the potential actions on the compensatory mechanisms of drugs aimed at reducing the actions of inflammatory mediators in the central nervous system (CNS).

Increased sympathetic nerve activity occurs in all phenotypes of heart failure (Parati and Esler, 2012). Initially, the increase in sympathetic nerve activity is compensatory but the sustained increase eventually is detrimental and worsens the prognosis (Kaye et al., 1995; Figure 1). The mechanisms mediating the increased sympathetic activity are not completely understood but the work in preclinical models of heart failure indicate that the central nervous system plays a major role, particularly nuclei such as the hypothalamic paraventricular nucleus (PVN), the nuclei of the lamina terminalis that lack a blood–brain barrier [e.g., the subfornical organ (SFO)] and medullary nuclei such as the rostral and caudal ventrolateral medulla (RVLM and CVLM, respectively; Xu et al., 2012).

The PVN projects to the intermediolateral cell column were sympathetic preganglionic motor neurons reside, and the PVN also sends collaterals to the RVLM neurons which send direct projections to the sympathetic preganglionic motor neurons as well (Shafton et al., 1998). Thus, the PVN has the anatomical connections that directly and indirectly influence sympathetic nerve activity. The PVN also receives inputs from the SFO and RVLM. An imbalance of excitatory and inhibitory inputs within the PVN has been suggested to contribute to the excessive sympathetic nerve activity in heart failure in rats (Zheng et al., 2011), suggesting these anatomical pathways play an important role. Peripheral afferent inputs that appear to contribute to the activation of the central pathways mediating the increased sympathetic outflow in heart failure include overactivity of the afferent renal nerves, overactivity of the carotid sinus afferents and angiotensin II (Schultz et al., 2015; Zheng et al., 2018).

### Characteristics of Heart Failure: Activation of the Renin–Angiotensin System

In heart failure, there is activation of the renin–angiotensin system which plays an active role in the remodelling of the heart and in fluid and electrolyte imbalance which are compensatory initially but eventually leads to worsening of the condition (Figure 1). Given the wide distribution of the renin–angiotensin system in the cardiovascular system and in the heart, it is not surprising that angiotensin-converting enzyme inhibitors and angiotensin receptor blockers have become key first line therapeutic interventions in heart failure.

The renin–angiotensin system is complex and consists of different receptor subtypes and, upon activation, they may initiate similar or even antagonistic responses, as is seen with the activation of type 1 (AT1R) and type 2 (AT2R) angiotensin receptors in various tissues (Kurdi et al., 2005). In addition to the classical ten amino acid peptide, angiotensin II, there are several angiotensin peptides (e.g., Ang 1-7, Ang 2-8 and Ang 1-9) produced by metabolism of angiotensin II by different enzymes. These peptides may have their own actions through binding to the classical or their own specific receptors such as in the case with Ang 1-7 that can bind AT1R, AT2R and the *mas* receptor (Iusuf et al., 2008). The latter may be particularly important for cardiac hypertrophy and fibrosis (Wang et al., 2017).

Adding to the complexity is the fact that components of the renin–angiotensin system are widely distributed and can induce local production of angiotensin (for example in the heart) as well as systemic production of angiotensin. Further, the presence of a central renin–angiotensin system means that locally produced angiotensin in the brain can also play a key role in cardiovascular disease and autonomic dysfunction. Additionally, centrally mediated actions can be elicited by peripherally produced angiotensin II acting on nuclei that lack a blood brain barrier, like the SFO (Wright and Harding, 2013).

Angiotensin II can elicit an increase in sympathetic nerve activity and this involves both central as well as peripheral sites of action. Several nuclei within the central nervous system, like the PVN, SFO and RVLM, have dense concentrations of angiotensin receptors and contribute to the increase in sympathetic nerve activity induced by angiotensin II and observed in heart failure (Zheng et al., 2009; Wang et al., 2014; Sharma et al., 2021). Thus, there exists an inter-relationship between the renin–angiotensin system and sympathetic nerve activation in heart failure. This inter-relationship may also exist with inflammation since angiotensin II can mediate inflammatory responses and could contribute to the increase in inflammation that is seen in heart failure.

### Characteristics of Heart Failure: Increased Inflammation

Markers of inflammation in the heart and in the circulation are observed in patients diagnosed with heart failure (Figure 1). Indeed, the levels of circulating cytokines are correlated with the severity of heart failure and prognosis (Rauchhaus et al., 2000; Braunwald, 2008). It has been known for some time that pro-inflammatory cytokines, for example tumour necrosis factor alpha (TNF-α), interleukin-1β, interleukin-2 and interleukin-4, can induce pulmonary oedema, ventricular contractility abnormalities and dysfunctional cardiac metabolism which can result in reduced cardiac function (Hegewisch et al., 1990; Bradham et al., 2002).

Circulating pro-inflammatory cytokines can also influence cardiac function further afield than just locally in the heart. More recent work has suggested that circulating pro-inflammatory cytokines can activate the sympathetic nervous system *via* activation of cells within the SFO. The effects of circulating pro-inflammatory cytokines on sympathetic nerve activity can be reproduced by direct microinjection of those pro-inflammatory cytokines into the SFO (Wei et al., 2015). The mechanisms involved are not clear but activation of the local renin–angiotensin system in the SFO and subsequent increases in prostaglandins and reactive oxygen species in the PVN and further cytokine production in the PVN appear to be involved (Wei et al., 2015). A critical role of the pro-inflammatory cytokine, TNF-α, in the SFO is further reinforced by the finding that the knockdown of the TNF-α receptor 1 in the SFO reduced the increase in sympathetic nerve activity normally observed in the myocardial infarction-induced model of heart failure (Yu et al., 2017). Despite the improved cardiac haemodynamics in the treated rats, cardiac function did not improve over the short observation period.

Thus, although abundant preclinical evidence would suggest that targeting the pro-inflammatory cytokines would be a useful therapy to treat heart failure, functional improvement in heart failure still appears elusive. To date, it has been very disappointing to see the results of clinical trials that have attempted to inhibit the actions of pro-inflammatory cytokines. Clinical trials targeting TNF-α using etanercept or infliximab in heart failure (e.g., RENAISSANCE, RECOVER, RENEWAL and ATTACH) were prematurely terminated due to the lack of beneficial outcomes or, in some instances, worse outcomes for patients (Chung et al., 2003; Mann et al., 2004). Although such trials have not provided the outcomes expected based on preclinical studies, there may still be some positive and encouraging signs emerging from more recent studies targeting other pro-inflammatory cytokines (CANTOS trial). In particular, the finding using Canakinumab to inhibit interleukin-1β function, showed a reduction in hospitalisations and mortality due to heart failure (Everett et al., 2018).

### What Are the Key Challenges?

It would appear perplexing that studies in animals strongly suggest a role of pro-inflammatory cytokines in the aetiology of heart failure, yet the outcomes of clinical trials using anti-inflammatory therapeutics have been decidedly unimpressive and have not provided support for such a view. What could explain this paradox? There are several possibilities that could be considered, and I would like to focus on two in this mini-review; Firstly, the target needs to be more specifically identified. Second, the inflammatory mediators in the CNS play a greater role, thus targeting these more specifically may be required.

### Targeting Specific Pro-Inflammatory Mediators and the Role of the CNS

The evidence to date indicates that pro-inflammatory mediators like TNF-α, interleukins, reactive oxygen species and angiotensin II are increased in heart failure. These increases occur locally within the myocardium, systemically (i.e., circulating in the blood) and within the central nervous system. Therapeutic agents like angiotensin-converting enzyme inhibitors and angiotensin II receptor blockers are front line therapy for heart failure, both in HFrEF and HFpEF. However, the evidence suggests they ameliorate the symptoms of HFrEF and have positive outcomes, but they have little or no positive influence on HFpEF. Anti-TNF-α treatments investigated in animal models of heart failure showed great promise; unfortunately, these hopes have not been fulfilled in clinical studies. But as noted earlier, there is a glimmer of hope from the recent findings in the CANTOS trial raising the possibility that specific targeting of interleukin-1β may be beneficial. This does raise the possibility that targeting specific pro-inflammatory mediators may be the way forward and clearly needs more work.

Another issue that needs to be addressed is the role of inflammatory mediators within the CNS, which may be playing a larger role than hitherto appreciated. Circumstantial evidence for this view arises from many studies. For example, TNF-α is increased in specific brain nuclei known to influence sympathetic nerve function (Kang et al., 2008). Direct microinjections of TNF-α into specific brain nuclei increases sympathetic nerve activity (Korim et al., 2019). and anti-TNF-α treatment reduces the abnormally elevated sympathetic nerve activity in an ischaemia-induced model of heart failure in rats (Guggilam et al., 2007). These studies suggest a key role for TNF-α within the brain in heart failure aetiology. This view is further supported by studies in which reducing the synthesis of TNF-α by decreasing the function of TNF-α-converting enzyme resulted in improved systemic haemodynamic variables while increased levels of the enzyme resulted in worse symptoms of heart failure in rats (Yu et al., 2019).

Interleukins in the brain are also important. The gene transfer of human interleukin-10 (a potent anti-inflammatory) using intraventricular administration in a rat model of heart failure resulted in amelioration of heart failure symptoms (Yu et al., 2007). Similarly, inhibition of pro-inflammatory cytokines in the brain using pentoxifylline reduced the neurohumoral excitation, that normally accompanies heart failure, in the coronary ligation model of heart failure in rats (Kang et al., 2008).

It should not be forgotten that there may exist a complex relationship between central and peripheral pro-inflammatory mediators. For example, elevated levels of circulating pro-inflammatory cytokines and angiotensin II can activate neurons in the SFO and result.

in an increased production of pro-inflammatory mediators within the brain. Pro-inflammatory processes in the brain may contribute to the sympathetic nerve dysregulation observed in heart failure; for example, increased levels of reactive oxygen species (Gao et al., 2004; Guggilam et al., 2007; Sharma and Patel, 2017). Inhibition of the production of reactive oxygen species in the brain ameliorated the autonomic dysfunction normally observed in ischaemia-induced heart failure in mice (Lindley et al., 2004). It is also noteworthy that increased production of reactive oxygen species can also be induced by angiotensin II (Gao et al., 2004). Thus, pro-inflammatory mediators could maintain the increased sympathetic nerve activity and contribute to the detrimental outcomes in heart failure. In this regard, it is interesting to note the findings of a recent Cochrane review on the impact of six different angiotensin-converting enzyme inhibitors on outcomes in patients with HFpEF. This review found little or no effect on all-cause and cardiovascular mortality and quality of life measures of angiotensin-converting enzyme inhibitors on this phenotype of heart failure (Martin et al., 2021). However, only one of the inhibitors used is known to cross the blood brain barrier leaving the question of whether targeting of pro-inflammatory mediators, like angiotensin within the CNS is beneficial in heart failure unanswered. Since attenuating the increase in pro-inflammatory cytokines in the CNS can ameliorate the increase in sympathetic nerve activity observed in heart failure, perhaps further studies which target specific peripheral and CNS pro-inflammatory mediators is needed (Figure 1).

### New Developments in Targeting Pro-Inflammatory Mediators in the CNS

From the preceding arguments, targeting specific pro-inflammatory mediators in the CNS may be a novel therapeutic approach to deal with the detrimental outcomes in heart failure and needs to be addressed. However, there is perhaps, a more critical issue, and that is related to the access of effective doses of the drugs to the site of action in the CNS. It is not possible to answer this easily because targeting the CNS for therapeutic intervention presents unique challenges due to the presence of the blood brain barrier and the ability to get drugs effectively to their site of action. Recent advances in delivery technology, however, are exciting (Vashist et al., 2018; Moura et al., 2019; Saeedi et al., 2019; Shahjin et al., 2020). Among the possibilities are (i) transient disruption of the blood brain barrier, (ii) direct microinjections into the cerebrospinal fluid, a rather invasive methodology which is unlikely to be welcomed by patients for regular ongoing treatment, (iii) extracellular vesicles and (iv) nanotechnology transporting techniques. The latter is particularly attractive and is advancing at a rapid rate (Saeedi et al., 2019).

Nanotechnology involving nanoparticles that contain two chemicals, one used as a diagnostic marker to allow for visualisation and tracking of the particle, and the second a therapeutic agent, have been described. An example is the recent targeting of microglia with such nanoparticles containing the anti-inflammatory drug, rolipram (Cahalane et al., 2020). In this *in vitro* study, microglia preferentially took up the nanoparticles compared to astrocytes. A promising result for future *in vivo* studies, given the role of microglia in inflammatory mediated processes within the brain.

Other studies have used specific cell/cell receptor targeting techniques to improve penetration into the CNS. For example, the use of liposomes containing anti-vesicular cell adhesion molecule-1, to target vascular endothelial cells *in vivo*, increased penetration into the CNS dramatically. This technique has been useful in reducing inflammation in the brain and restoring integrity of the blood brain barrier in a model of brain inflammation (Marcos-Contreras et al., 2020). A similar targeting technique has been used *in vivo* to alter the behaviour of mice in studies of depression. Using negatively charged liposomes containing trefoil factor 3, because of its anti-depressant functions, resulted in improved mobility and reduced immobility in behavioural tests in mice (Qin et al., 2014). A similar positive outcome has been observed using a more targeted approach in which the liposomes were modified so that their affinity for monocytes was markedly increased (Qin et al., 2015). Other strategies utilised include stimuli sensitive nanoparticles. These exciting methodologies utilise environmentally sensitive nanoparticles that can change their shape according to the micro-environment, for example pH. redox capability, presence of enzymes (Lee and Thompson, 2017; Mohamed et al., 2019). Together, these new innovative developments in nanotechnology increase the likelihood that penetration into the CNS will improve dramatically in the future and suggests that a more targeted approach to dealing with inflammatory processes in the brain is on the way.

In summary, heart failure is a growing medical problem which will place a continued heavy burden on health budgets. The level of ejection fraction has normally determined the phenotype of heart failure, but most patients diagnosed with heart failure have ejection fractions less than 50%. Although the underlying aetiology of heart failure differs according to the phenotype, as suggested by the different responses to pharmacological treatment, there are some common characteristics observed in patients with heart failure. These include an increased sympathetic nerve activity, an activated renin–angiotensin system, and inflammation. The mechanisms mediating the increased sympathetic activity are not completely understood but the central nervous system plays a major role. Activation of the renin–angiotensin system plays an active role in the remodelling of the heart and in fluid and electrolyte imbalance which are compensatory initially but eventually leads to worsening of heart failure. The presence of a central renin–angiotensin system means that locally produced angiotensin in the brain may also play a key role in autonomic dysfunction seen in heart failure. Markers of inflammation in the heart and in the circulation are observed in patients diagnosed with heart failure. Circulating pro-inflammatory cytokines can also influence cardiac function further afield than just locally in the heart. More recent work has suggested that circulating pro-inflammatory cytokines can also activate the sympathetic nervous system. Strong preclinical evidence would suggest that targeting the pro-inflammatory cytokines would be a useful therapy to treat heart failure. However, most clinical studies do not support such a view, though there may be a glimmer of hope in recent studies (CANTOS trial). If specific pro-inflammatory mediators in the CNS contribute to the detrimental outcomes in heart failure, I suggest that a key problem is associated with access of effective doses of the drugs to the site of action in the CNS. The recent advances in delivery technology, particularly nanotechnology transporting techniques, are evolving at a rapid rate and may provide exciting future technology to investigate the role of specific pro-inflammatory mediators as novel targets within the brain in the treatment of heart failure.

## Author Contributions

The author confirms being the sole contributor of this work and has approved it for publication.

## Conflict of Interest

The author declares that the research was conducted in the absence of any commercial or financial relationships that could be construed as a potential conflict of interest.

## Publisher’s Note

All claims expressed in this article are solely those of the authors and do not necessarily represent those of their affiliated organizations, or those of the publisher, the editors and the reviewers. Any product that may be evaluated in this article, or claim that may be made by its manufacturer, is not guaranteed or endorsed by the publisher.
